# Mitochondrial myopathy plus due to the variant m.586G > A in *MT-TF*

**DOI:** 10.1016/j.ymgmr.2019.100519

**Published:** 2019-09-11

**Authors:** Josef Finsterer

**Affiliations:** Krankenanstalt Rudolfstiftung, Messerli Institute, Postfach 20, 1180 Vienna, Austria

**Keywords:** Myopathy, mtDNA, Mitochondrial, Lactic acidosis, Headache, Oxidative phosphorylation

Letter to the Editor,

With interest we read the article by Barcia et al. about a 14 years-old male with mitochondrial myopathy due to the variant m.586G > A in *MT-TF* [1]. The variant most likely occurred spontaneously, as either of the parents manifested clinically and as the mother did not carry the culprit variant. We have the following comments and concerns.

We do not agree that the presented patient had no central nervous system (CNS) involvement [1]. Phenotypic manifestations of the m.586G > A variant were not restricted to mitochondrial myopathy [1]. The patient had quadru-spasticity, and headache, both manifestation clearly indicating CNS involvement. We should know if cerebro-spinal fluid (CSF) lactate was elevated upon examination of the CSF or upon magnetic resonance spectroscopy (MRS). Concerning headache we should know if it was classified as tension headache, migraine, migraine-like headache, or cluster headache, all types of headache that had been reported in association with a MID [2].

Concerning vomiting after exercise we should know if this was due to exercise-induced lactic acidosis, frequently occurring in patients with mitochondrial myopathy. Thus, we should know if elevated resting lactate further increased upon exercise or upon mild exercise below the lactate threshold in the context of the lactate stress test [3]. Since MID patients may also manifest with primary involvement of the intestines [4], we should know if the patient had undergone gastroscopy or if there were other gastro-intestinal manifestations of the MID as well. We should also know if vomiting was associated with headache, as MID patients frequently develop migraine-like headache, being associated with gastro-intestinal compromise.

Since most MIDs manifest with clinically manifesting or subclinical multisystem involvement [5], prospective investigations for multi-organ involvement should be initiated.

Overall, the interesting patient had definitively CNS involvement and most likely exercise-induced lactic acidosis. Type and treatment of headache need to be specified.

## Funding

No funding was received.

## Author contribution

JF: design, literature search, discussion, first draft, critical comments.

Informed consent: was obtained.

The study was approved by the institutional review board.

## Declaration of Competing Interest

There are no conflicts of interest.
